# Development of Electrochemiluminescent Serology Assays to Measure the Humoral Response to Antigens of Respiratory Syncytial Virus

**DOI:** 10.1371/journal.pone.0153019

**Published:** 2016-04-12

**Authors:** Sarah V. Maifeld, Bodrey Ro, Hoyin Mok, Marla Chu, Li Yu, Ryan Yamagata, Tansy Leonardson, Vera Chio, Bandita Parhy, Samuel Park, Marcia Carlson, Shushil Machhi, Nancy Ulbrandt, Ann R. Falsey, Edward E. Walsh, C. Kathy Wang, Mark T. Esser, Fengrong Zuo

**Affiliations:** 1 Applied Immunology and Microbiology, MedImmune, Mountain View, California, United States of America; 2 Non-clinical Biostatistics, MedImmune, Gaithersburg, Maryland, United States of America; 3 Vaccine and Analytical Sciences, MedImmune, Mountain View, California, United States of America; 4 Purification Process Sciences, MedImmune, Gaithersburg, Maryland, United States of America; 5 Cell Culture and Fermentation Sciences, MedImmune, Gaithersburg, Maryland, United States of America; 6 Department of Infectious Disease and Vaccines, Gaithersburg, California, United States of America; 7 Department of Medicine, University of Rochester School of Medicine and Dentistry, and Department of Medicine, Rochester General Hospital, Rochester, New York, United States of America; 8 Translational Medicine, MedImmune, Gaithersburg, Maryland, United States of America; University of Iowa, UNITED STATES

## Abstract

Sensitive and precise serology assays are needed to measure the humoral response to antigens of respiratory syncytial virus (RSV) following natural infection or vaccination. We developed and evaluated a collection of electrochemiluminescent (ECL) serology assays using four RSV antigens (F, N, Ga and Gb). To assess the merits of ECL technology, the four ECL serology assays were evaluated using a well-characterized “gold standard” panel of acute and convalescent serum samples from fifty-nine RSV-positive and thirty RSV-negative elderly subjects (≥65 years old). The combined results from the four ECL assays demonstrated good concordance to the “gold standard” diagnosis, reaching 95% diagnostic sensitivity and 100% diagnostic specificity. Additionally, a combination of ECL assays provided higher diagnostic sensitivity than a commercially available diagnostic ELISA or cell-based microneutralization assay. In summary, these data demonstrate the advantages of using ECL-based serology assays and highlight their use as a sensitive diagnostic approach to detect recent RSV infection in an elderly population.

## Introduction

Respiratory syncytial virus (RSV) is a worldwide cause of severe lower respiratory tract infections. Two distinct antigenic subtypes, RSV A and B, circulate independently or simultaneously to cause illness during annual RSV seasons [1]. Morbidity and mortality resulting from RSV infection are common in high-risk populations such as infants and young children [2], the elderly and individuals of all ages with cardiopulmonary disease or compromised immune systems [3]. RSV infection is recognized as the primary cause of hospitalization for acute lower respiratory tract infection among infants worldwide, resulting in an estimated 2.1 million children receiving medical care each year in the U.S. [2]. Among adults over the age of 65, RSV infection contributes to over 170,000 hospitalizations and 14,000 deaths annually in the U.S [3]. Palivizumab, a neutralizing monoclonal antibody which recognizes the RSV fusion (F) protein, is used for prevention of RSV disease in high-risk infants [4]; however, no prophylactic treatment such as a vaccine or monoclonal antibody is available for other susceptible populations [5].

Sensitive and specific assays to detect recent RSV infection are useful to understand the incidence of RSV infection and potentially identify a correlate of protection from epidemiology studies and vaccine clinical trials [6]. Although serology has been shown to be a more sensitive diagnostic approach than viral culture or RT-PCR in adult populations [7], existing serology assays, such as ELISA or cell-based microneutralization assays, have limitations. Colorimetric ELISA tests have a narrow dynamic range while cell-based microneutralization assays may have higher variability and are more labor intensive. For these reasons, we evaluated Meso Scale Discovery (MSD)’s electrochemiluminescence (ECL) technology platform for its reported wide dynamic range, improved analytical sensitivity and reduced non-specific background signal.

Of the eleven proteins encoded by the RSV genome, we selected the fusion (F), nucleocapsid (N) and attachment (G) proteins for assay development using ECL technology. Both F and G antigens elicit neutralizing antibodies that can provide protection against subsequent infection [8], and RSV vaccines frequently include or express these antigens [9–11]. The use of F, N and G antigens to measure serum antibody levels from RSV exposure is well-documented [12–16]. Although the amino acid sequences of F and N are highly conserved between RSV A and B subtypes [17, 18], the sequence of G differs dramatically and provides the principle source of antigenic variation among circulating strains [19–23]. In order to measure G-specific antibodies regardless of the infecting strain’s subtype, we included G antigen from both RSV subtypes (Ga and Gb) as part of our diagnostic strategy.

Four ECL assays (F, N, Ga and Gb IgG) were developed and evaluated for analytical and diagnostic performance [24]. To evaluate the diagnostic sensitivity and specificity of the four ECL assays, we assembled a well-characterized, “gold standard” panel of acute and convalescent serum samples from eighty-nine elderly (≥65 years old) participants of an RSV surveillance study [3]. Our results demonstrate that RSV antigen-specific serology assays using ECL technology have several advantages and provide an improved method to detect recent RSV infection in an elderly population.

## Materials and Methods

### Reagents

RSV antigens were expressed and purified to >90% purity, as determined by SDS-PAGE. Fifty milligrams (50 mg) of a soluble and post-fusion form of the F antigen from the RSV A2 strain was expressed in Chinese Hamster Ovary (CHO) cells and affinity purified with an anti-RSV F monoclonal antibody (palivizumab, MedImmune, Gaithersburg, MD) [25]. The RSV F protein sequence is available in the GenBank database (http://www.ncbi.nlm.nih.gov/GenBank) under accession number KJ155694. One hundred milligrams (100 mg) of full length nucleocapsid protein N from the RSV A2 strain containing a GST tag with a thrombin cleavage site was expressed in *Rosetta (DE3) E*. *coli* using a pGEX-4T3 vector. The protein was purified using a GSTrap^TM^ column (GE Healthcare) and a Butyl HP HiTrip^TM^ column (GE Healthcare) to remove excess nucleic acid. Ten milligrams (10 mg) of the extracellular domain of glycoprotein G from RSV A strain rsb1734 (95% homology to A2) was purchased from Sino Biological Inc (Beijing, China). The ectodomain of RSV glycoprotein G from RSV B strain B9320 was expressed with an N-terminal histidine tag in 293F cells. The supernatant containing the RSV Gb protein was affinity purified by Ni^2+^ chromatography using a Hi-Trap column (GE Healthcare), followed by size exclusion chromatography using a Superdex 200 column (GE Healthcare) to remove protein aggregates. Motavizumab was supplied by MedImmune (Gaithersburg, MD).

### Human serum samples

Archived acute and convalescent serum samples from elderly patients (≥65 years old) were selected from an RSV surveillance study [3]. Written informed consent was obtained from all participants prior to enrollment in the study and approved by the University of Rochester Institutional Review Board. The use of sera for immunologic and diagnostic testing in this study was specified by the written informed consent and approved by the University of Rochester Institutional Review Board. Twenty-nine subjects were categorized as “RSV A-positive” after nasopharyngeal samples tested positive for RSV A by RT-PCR and paired serum samples showed a ≥4-fold rise in serum titer by at least one ELISA assay (RSV F, Ga or Gb IgG). Thirty subjects were categorized as “RSV B-positive” after nasopharyngeal samples tested positive for RSV B by RT-PCR and paired serum samples showed a ≥4-fold rise in serum titer by at least one ELISA assay (RSV F, Ga or Gb IgG). Another 30 subjects, categorized as “RSV-negative,” were from patients with clinical respiratory symptoms but without laboratory evidence of recent RSV infection as demonstrated by negative RT-PCR results and paired serum samples showing a <4-fold rise in serum titer by ELISA (RSV F, Ga or Gb IgG). An additional 49 subjects tested negative by RT-PCR for either RSV A or B yet had paired serum samples showing a ≥4-fold rise in at least one ELISA assay. Thirty pediatric serum samples were purchased from Bioreclamation (Hicksville, NY). An adult human plasma sample was purchased from AllCells (Emeryville, CA) and subsequently used as the assay control for development and control trending purposes.

### RSV ECL assays

Each MSD plate contained up to ten serially diluted samples, one serially diluted assay control and eight blank wells in which no sample–only dilution buffer–was added. Duplicate samples were run on separate plates. A generalized protocol for the RSV F, N, Ga and Gb IgG ECL assays follows. Specific values of coated antigen and concentrations of goat anti-human IgG SULFO-TAG antibody are listed in Table 1. RSV antigen was diluted in PBS (0.2–0.6 μg/ml) and added to standard MSD 96-well plates (50 μL/well). Plates were sealed and stored at 4°C overnight or up to 3 days before use. Plates were blocked with 5% BSA in PBS (200 μL/well) and gently shaken for one hour at room temperature. Plates were washed using an AquaMax automated plate washer (Molecular Devices, Sunnyvale, CA). Human serum samples were prepared at an appropriate dilution (1:20 for pediatric samples or 1:1,000 for elderly samples) in dilution buffer (5% BSA, 2.5% CHAPS, 300 mM NaCl and 0.5% Tween-20 in PBS lacking calcium and magnesium). A minimum of 5 μL of sample was used to prepare the starting dilution. Samples were serially diluted in dilution buffer according to an 8-point, 3-fold dilution scheme using a BravoSRT automated liquid handler (Agilent Technologies, Santa Clara, CA). The serially diluted samples (50 μL/well) were then transferred to MSD plates using the BravoSRT liquid handler followed by room temperature incubation with gentle shaking for one hour. After washing the plates, goat anti-human IgG SULFO-TAG antibody (Meso Scale Discovery, Gaithersburg, MD) in dilution buffer (0.375–1.25 μg/ml) was added (50 μL/well) to the plates. Plates were gently shaken at room temperature for one hour. After washing the plates, 2X MSD Read Buffer T with surfactant (Meso Scale Discovery, Gaithersburg, MD) was added (150 μL/well). Plates were scanned immediately on an MSD Sector Imager 6000. Relative light units (RLU) from each well were enumerated by MSD software, log_10_-transformed and plotted versus the 8-point serial dilution scheme. The RSV antigen-specific IgG antibody titer of each serum sample was calculated using a simple linear regression and a cutoff fifteen-times the mean background signal of the plate. Final serum titers were calculated as the mean value from duplicate plates. Seroresponse following natural infection was defined as ≥4-fold rise of serum titer between acute and convalescent samples.

**Table 1:** RSV ECL assay conditions.

	F	N	Ga	Gb
Antigen coating (ng)	10	30	20	10
SULFO-TAG antibody (μg/ml)	0.5	0.5	0.375	0.375

### RSV F/G IgG diagnostic ELISA

Samples were tested using an FDA-exempt, RSV diagnostic ELISA kit (catalog number IB79280, IBL-America, Minneapolis, MN). Samples were appropriately diluted (e.g. 1:100 or 1:10,000) and run in the assay according to the manufacturer’s instructions. Serum titers were calculated based on the kit’s standard calibration curve using a 4-parameter logistic fit. Acute and convalescent serum samples were diluted until the acute sample fell within the assay range and at least 4-fold below the ULOQ (upper limit of quantitation) of the calibration curve. All sample pairs showing at least a 4-fold rise in ELISA titer were assigned a fold change value of 4. The product literature from the manufacturer (IBL-America) describes the interassay precision of the RSV F/G IgG ELISA as 10.7% and maximum inter-lot precision as 12.1%.

### RSV-GFP A2 microneutralization assay

Heat-inactivated serum samples were serially diluted in DMEM (containing 2% L-Glutamine, 1% Pen/Strep and 1% NEAA) by a BravoSRT liquid handler. Equal volumes of the diluted sera were mixed with recombinant green fluorescent protein (GFP)-tagged RSV A2 virus at a concentration of 500 plaque-forming units per well. This virus-serum mixture was incubated for one hour at 33°C and 5% CO_2_. The virus-serum mixture was transferred to 96-well clear bottom microplates containing Vero cells grown to confluency. Each cell plate contained up to ten serially diluted samples, one serially diluted assay control (pooled adult human serum) and eight negative control wells in which no sample–only dilution buffer–was added. The infected cell plates were incubated for 22 hours at 33°C and 5% CO_2_. The cell plates were washed with PBS and fluorescent foci were enumerated using an IsoCyte^TM^ Reader (Blueshift Biotechnologies, Sunnyvale, CA). Neutralization titers were calculated using a four-parameter logistic model and reported as log_2_ IC_50_ values. Trending of the RSV microneutralization assay control showed a 28% CV from experiments (n = 1,567) conducted over 793 days by six analysts.

### Statistical analysis

For experiments using motavizumab, the 4-parameter logistic model was used to determine the limit of detection (LOD) and the linear range for each assay [26]. Statistical significance was determined using a two-sample t-test between age groups. The numbers of true positives (TP), true negatives (TN), false positives (FP) and false negatives (FN) were used to calculate diagnostic sensitivity and specificity. Sensitivity was calculated as 100*TP/(TP+FN); specificity was calculated as 100 * TN/(TN+FP). Assay variability was estimated using a variance component model and reported as the percent coefficient of variability (% CV) [27]. Endpoint titers and the standard deviation of the log2 endpoint titers of the RSV ECL assay control were used in the calculations. Statistical analysis was performed using GraphPad Prism 6 software and SAS 9.0.

## Results

### Development and characterization of four ECL assays

**Fig 1:**
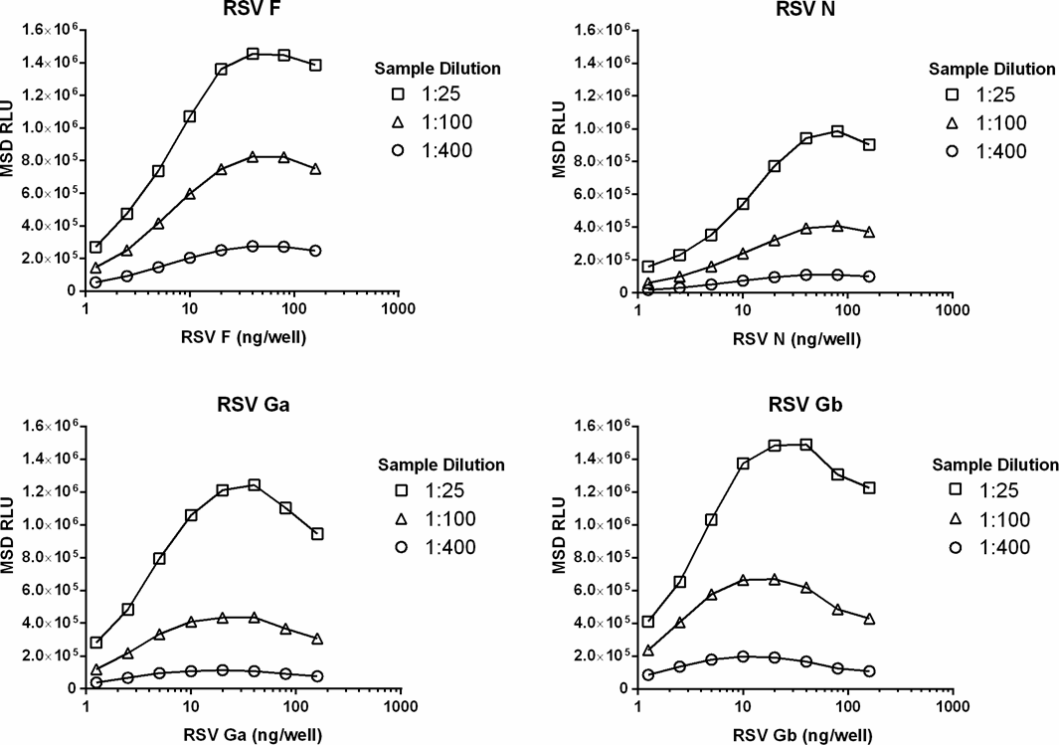
Representative example of an antigen titration experiment to determine optimal coating density. For each antigen, a stock solution was serially diluted and coated onto 96-well MSD plates. In this example, three dilutions of the assay control (MN log_2_ IC_50_ titer = 9.9) were tested at a fixed concentration of goat anti-human IgG SULFO-TAG antibody (0.5 ug/ml). Symbols represent mean values from eight replicates.

We developed four ECL assays (RSV F, N, Ga and Gb IgG) to measure antigen-specific IgG responses following natural infection or vaccination with RSV vaccine clinical candidates. The optimal assay conditions, including the amount of coated antigen and concentration of SULFO-TAG antibody, were determined using the assay control, an individual human sample with a known RSV microneutralization titer (log_2_ IC_50_ = 9.9). Antigen titration experiments using three dilutions (1:25, 1:100 and 1:400) of the assay control revealed a significant “hook effect” at higher coating densities (Fig 1). The optimal assay conditions were those that maximized each assay’s linear range and minimized non-specific signal in background wells (Table 1).

**Fig 2:**
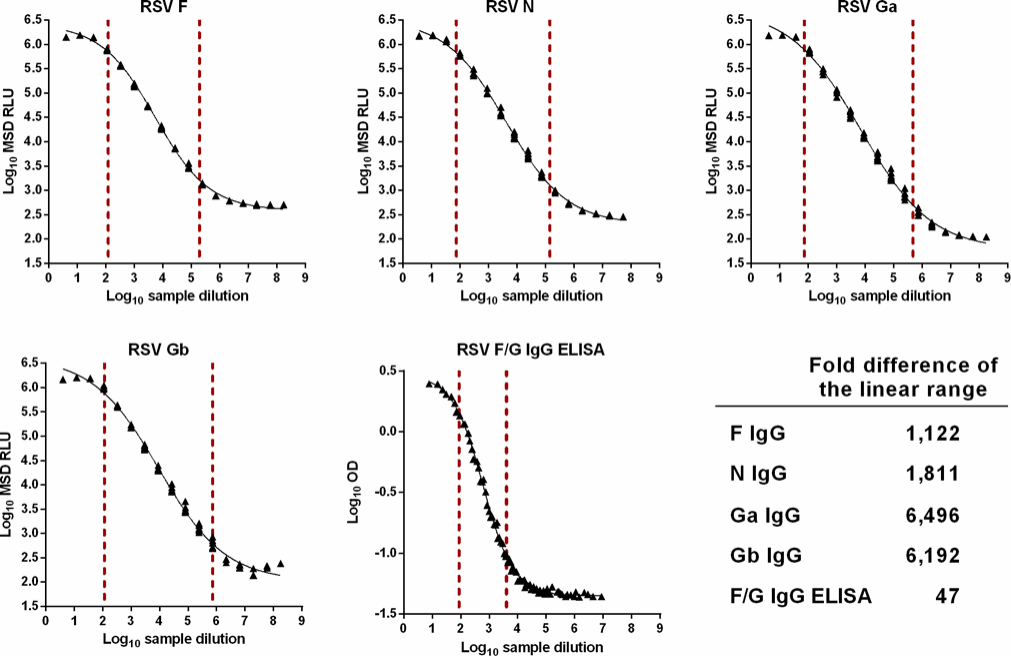
Linear range comparison between RSV ECL assays and a diagnostic RSV F/G ELISA. The assay control was used to illustrate the linear range of the four ECL assays and the diagnostic F/G ELISA. Linearity samples were prepared from the assay control and serially diluted before addition to the MSD or ELISA plates. MSD RLU or ELISA OD (optical density) values were log_10_-transformed and plotted versus the log_10_-transformed sample dilution. The upper and lower limits of the linear range were calculated using a 4-parameter logistic model and marked by dashed red lines. The fold difference was calculated for each assay as the ratio between limits of the linear range.

The linear range of each ECL assay was compared to an FDA-exempt, diagnostic F/G ELISA using the assay control as a representative sample (Fig 2). In contrast to the colorimetric TMB readout used in the diagnostic ELISA, the ruthenium-labeled detection antibody in the ECL assays can undergo multiple excitation cycles and generate a wider dynamic range than the diagnostic ELISA [28]. The Ga IgG ECL assay exhibited the widest linear range (6,496-fold) while the diagnostic ELISA showed the narrowest (47-fold).

Next, the analytical sensitivity of the F IgG ECL assay was directly compared to the diagnostic ELISA using motavizumab, a humanized monoclonal antibody which binds to a neutralizing epitope on RSV F (Table 2, S1 Fig) [29]. The binding of motavizumab was linear across a wider range in the F IgG ECL assay (39.8–35,481.3 pg/ml) than the diagnostic ELISA (851.1–26,302.7 pg/ml). The lowest detectable concentration of motavizumab was approximately 40-fold lower in the F IgG ECL assay than the diagnostic ELISA, further confirming the benefits of ECL detection technology.

**Table 2:** Analytical sensitivity using an anti-RSV F neutralizing monoclonal antibody (motavizumab).

	Limit of detection (pg/ml)	Lower limit of quantitation (pg/ml)	Upper limit of quantitation (pg/ml)	Fold Difference of the Linear Range
RSV F IgG	6.2	39.8	35,481.3	891.5
RSV F/G IgG ELISA	245.4	851.1	26,302.7	30.9

Performance of the assay control was evaluated for each ECL assay using experiments from at least four different days and two analysts (S2 Fig). The percent coefficient of variability (% CV) of the RSV F, N, Ga and Gb IgG assays was 14.4%, 12.8%, 19.3% and 18.5%, respectively. These data show that the ECL assays are precise and suitable to measure the humoral response in human sera.

### RSV-specific IgG antibody levels in infants (2–24 months) and elderly (≥65 years old)

**Fig 3:**
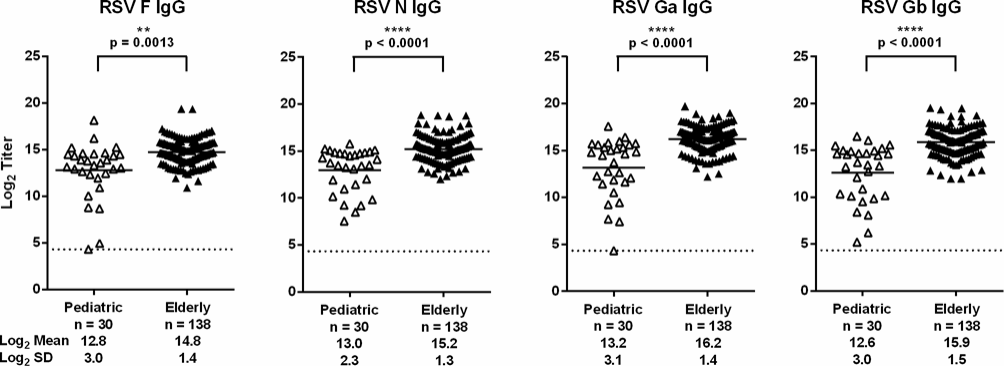
Comparison of infant and elderly serum samples in four ECL assays. The mean log_2_ titer is represented for each group. The dashed line represents the lower limit of quantitation (log_2_ 20 = 4.32). Significance between age groups was determined using a two sample t-test.

The RSV F, N, Ga and Gb IgG ECL assays were used to measure and compare RSV antigen-specific IgG antibody levels in infant and elderly populations. Thirty serum samples from infants (2–24 months) and 138 acute-phase serum samples from elderly (≥65 years old) patients were tested in each of the four assays (Fig 3). Overall, antibody levels to RSV antigens varied widely. The mean serum titer was higher in the elderly group than the infant group for each assay. The infant population exhibited a wider range of serum titers, with the largest difference (>14,000-fold, 13.8 log_2_) observed in the RSV F IgG ECL assay. As these results demonstrate, the wide dynamic range of ECL assays is well-suited to measure the variation of RSV-specific IgG antibody levels in infant and elderly serum.

### Diagnostic performance of the RSV ECL assays in an elderly population

**Fig 4:**
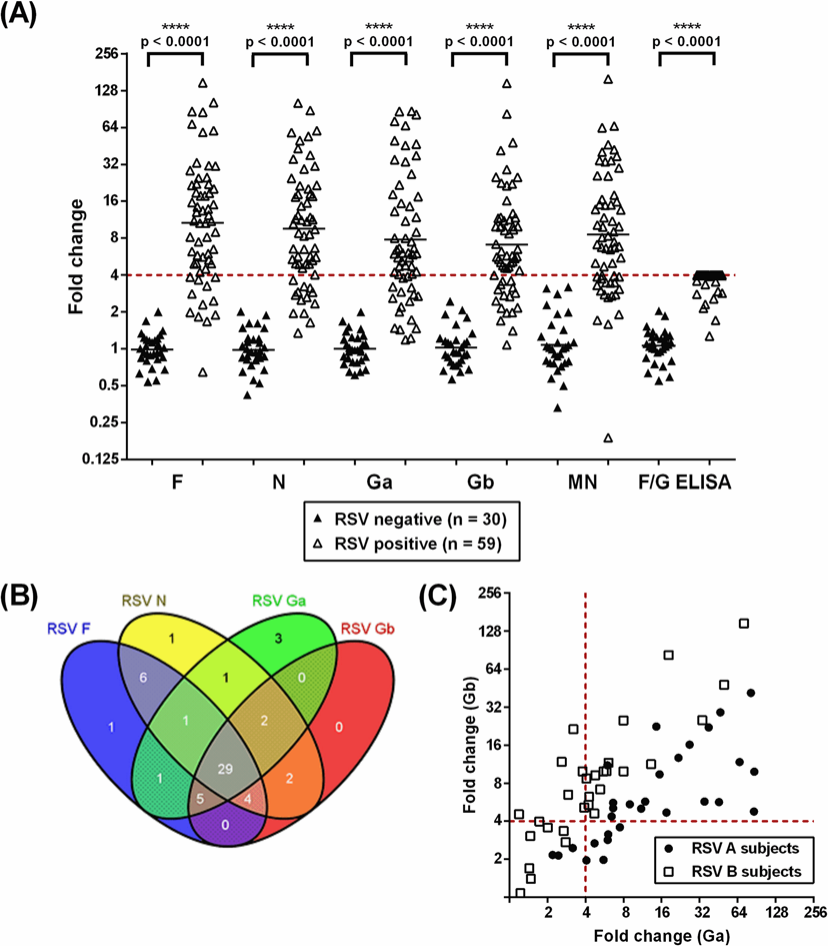
Seroresponse of naturally infected elderly subjects. (A) The fold change of serum titers between acute and convalescent serum samples from elderly subjects (59 RSV-positive, 30 RSV-negative) was measured by four ECL assays, a microneutralization assay and a diagnostic ELISA. A four-fold rise in serum titer, marked by a red dashed line, was used to indicate seroresponse following natural infection. Due to the limited dynamic range of the diagnostic F/G ELISA, acute and convalescent serum samples were diluted until the acute sample fell within the assay range and at least 4-fold below the ULOQ. To simplify the testing and analysis, all sample pairs showing at least a four-fold rise in ELISA titer were assigned a fold change value of four. (B) A Venn diagram illustrates the seroresponse profiles to RSV F, N, Ga and Gb from RSV-positive elderly subjects (n = 59), as measured by ECL assays. A ≥4-fold rise in serum titer was used to indicate seroresponse following natural infection. Three subjects did not show a ≥4-fold rise in any ECL assay. The Venn diagram was prepared by Venny software http://bioinfogp.cnb.csic.es/tools/venny/index.htm (C) Scatterplot showing the fold change between acute and convalescent serum pairs of RSV A (n = 29) and RSV B (n = 30) positive elderly subjects, as measured by RSV Ga and Gb IgG ECL assays.

The RSV F, N, Ga and Gb IgG ECL assays were used in a diagnostic proof-of-concept study to identify recent RSV infections in eighty-nine elderly patients (≥65 years old) enrolled in an RSV surveillance study. A “gold standard” panel of acute and convalescent serum pairs from 59 RSV-positive patients and 30 RSV-negative patients were tested in the four ECL assays, a cell-based RSV microneutralization assay and an F/G IgG diagnostic ELISA assay (Fig 4A). Following the historical precedent, a ≥4-fold rise between acute and convalescent serum titers was considered a biomarker of RSV infection [30]. The ability of each assay to correctly identify RSV-positive and RSV-negative subjects against the “gold standard” was evaluated (Table 3). All assays demonstrated excellent diagnostic specificity (100%) by correctly identifying the thirty RSV-negative individuals with zero false positives. The F IgG ECL assay demonstrated the highest diagnostic sensitivity (80%) by identifying 47 of the 59 RSV-positive individuals. The F IgG ECL assay performed slightly better than the diagnostic ELISA containing both F and G antigens (46 subjects, 78% sensitivity). The microneutralization assay had the lowest diagnostic sensitivity (68%), identifying 40 of the 59 RSV-positive individuals.

**Table 3:** Comparison of diagnostic sensitivity of RSV serology assays ^a^,^b^.

		# of subjects (total = 59)	Sensitivity (%)
**Existing serology assays**	**Microneutralization (MN)**	40	68
	**F/G ELISA**	46	78
**One ECL assay**	**F**	47	80
	**N**	46	78
	**Ga**	42	71
	**Gb**	42	71
**Combination of ECL assays**	**F or N**	53	90
	**F or Ga**	53	90
	**F or Gb**	51	86
	**N or Ga**	55	93
	**N or Gb**	51	86
	**Ga or Gb**	48	81
	**F or Ga or Gb**	55	93
	**N or Ga or Gb**	55	93
	**F or N or Ga or Gb**	56	95
	**F or N or Ga or Gb or MN**	56	95

^a^ Analysis of 59 RSV-positive elderly subjects

^b^ Seroresponse determined by a ≥4-fold rise in serum titer

Seroresponse to the four RSV antigens, defined by a ≥4-fold rise in antibody titer, varied in the RSV-positive subjects (Fig 4B). Twenty-nine individuals showed seroresponse in all four ECL assays; five subjects showed seroresponse in just one assay (F: 1; N: 1; Ga: 3). Three subjects did not show seroresponse in any of the four ECL assays, although two of these subjects showed ˃3-fold rise in the Ga or Gb IgG assays. Seroresponse to RSV antigens following natural infection varied by individual, and the combination of results from more than one ECL assay increased the diagnostic sensitivity. Pairing the F IgG ECL assay with either the N or Ga assay increased the diagnostic sensitivity from 80% to 90%. Fifty-five RSV-positive subjects showed seroresponse in either the N or Ga IgG ECL assays (93% sensitivity). The combined results from three ECL assays, such as F, Ga and Gb or N, Ga and Gb, identified the same number yet slightly different populations of RSV-positive subjects (55, 93% sensitivity). Finally, the combination of the four ECL assays (F, N, Ga and Gb) identified 56 of the 59 RSV-positive subjects (95% sensitivity).

Homosubtypic and heterosubtypic antibody responses were examined in the Ga and Gb IgG ECL assays based on the subtype of infecting virus (Fig 4C). Elderly subjects with RSV A or B infections generated significant levels of cross-reactive antibodies to G protein of either subtype. Of the RSV A-positive subjects (n = 29), twenty-six showed seroresponse in the Ga assay (90%). Of those 26 elderly subjects, 20 showed seroresponse in the Gb assay. A similar trend was observed for the 30 RSV B-infected subjects. Of the 22 elderly subjects showing ≥4-fold rise in Gb IgG titer (73%), 16 subjects showed seroresponse in the Ga assay. Overall, the fold change was greater when the antigen of the G assay was homologous to the infecting strain. The mean fold change from paired serum samples from RSV A-infected elderly subjects was higher in the Ga assay than the Gb assay (13.1 versus 6.2, respectively). Similarly, the mean fold change from paired serum samples from RSV B-infected subjects was greater in the Gb assay than the Ga assay (8.1 versus 4.7, respectively).

## Discussion

The development of sensitive and specific serology assays to measure levels of RSV-specific antibodies elicited by vaccines or natural infection required the appropriate choice of RSV antigens and assay platform. Recombinantly expressed and purified RSV viral proteins (F, N, Ga and Gb) were selected based on their documented immunogenicity following natural infection [12–16], relevance to vaccine clinical candidates [31–33] and sequence homology between subtypes [17,18, 20]. Because levels of RSV F, N, Ga and Gb-specific antibodies can vary widely in individuals and in response to infection or vaccination, we chose MSD’s ECL assay platform for its wide dynamic range, minimal non-specific background signals and low limits of detection. The development of four RSV ECL assays (F, N, Ga and Gb IgG) highlighted the advantages of ECL technology over a commercially available, diagnostic F/G ELISA assay using a colorimetric readout. When the assay control was used as a representative sample, the ECL assays had a linear range 23- to 138-times wider than the diagnostic ELISA. In the absence of a truly RSV negative serum, an RSV F-specific neutralizing monoclonal antibody (motavizumab) was spiked into PBS to compare the analytical sensitivity of the F IgG ECL assay to the diagnostic ELISA. ECL detection lowered the limit of detection for motavizumab nearly 40-fold.

A survey of RSV F, N, Ga and Gb-specific IgG antibody levels in infant and elderly serum illustrated the benefits of using ECL assays with a wide dynamic range. The elderly group exhibited a higher mean serum titer than the infant group in each of the ECL assays. This observation was not unexpected since immunity following primary infection is incomplete and subsequent infections occur throughout life [34,35]. The infants exhibited a wider range of serum titers possibly due to the waning presence of maternal antibodies and the response to primary or recurrent infections [36–38].

The four ECL assays, a cell-based microneutralization assay and an F/G IgG diagnostic ELISA assay were evaluated in a diagnostic proof-of-concept study using a well-characterized “gold standard” panel of acute and convalescent phase serum samples from eighty-nine elderly subjects. Fifty-nine subjects had documented RSV infection (i.e. RSV-positive) while an additional thirty subject were not infected by RSV (i.e. RSV-negative). The combined results from the four ECL assays demonstrated good concordance to the “gold standard” diagnosis (95% sensitivity, 100% specificity) and surpassed the diagnostic sensitivity of an RSV microneutralization assay or a diagnostic ELISA. The F IgG ECL assay demonstrated the highest diagnostic sensitivity (80%), performing better than the microneutralization assay (68% sensitivity) and the diagnostic F/G ELISA (78% sensitivity). The lower diagnostic sensitivity of the microneutralization assay may be partly explained by the assay’s measurement of functional antibodies that neutralize infectivity rather than the functional and non-functional antibodies measured by the ECL assays and the diagnostic ELISA [39,40]. A ≥4-fold rise in RSV antigen-specific antibody level for at least one ECL assay was demonstrated in 95% of the elderly subjects. Notably, the diagnostic specificity remained excellent (100%) when the seroresponse threshold was lowered to ≥3-fold rise in serum titer. At this threshold, 58 of the 59 RSV-positive subjects were identified (98% sensitivity). The lack of complete concordance to the “gold standard” panel could be due to repeated freeze-thaw cycles or prolonged storage of the samples.

Because the paired serum samples in the “gold standard” panel came from subjects previously categorized as RSV-positive or negative by both RT-PCR and ELISA tests, the sample panel provided a strong benchmark for our diagnostic proof-of-concept study. Future work may expand on the diagnostic capabilities of ECL serology assays by analyzing samples from participants with positive RT-PCR results but negative ELISA results. A previous study showed lower baseline levels of serum IgG antibodies to F, Ga and Gb in subjects who subsequently became RSV infected than age-matched controls who did not develop RSV infection [41]; however, there was no significant difference in titers of acute phase sera from RSV-positive or negative subjects selected for our study. For the samples used in this study, acute phase serum samples collected upon hospitalization may not have accurately reflected the baseline serum status since levels of antibodies in the elderly can rise rapidly following reinfection with RSV [42]. Although group- and strain-specific responses to G antigen have been observed in infants and children [43–45], the Ga and Gb assays measured substantial cross-reactive antibodies in serum from RSV A and B-infected elderly subjects [46]. This observation can likely be attributed to serum antibodies directed toward a stretch of 13 amino acids within the G protein’s central unglycosylated region that is universally conserved in all clinical isolates [19].

In summary, we have developed a collection of four sensitive and precise serology assays using ECL technology to measure IgG antibody responses following exposure to antigens of RSV. Previously, we used the F IgG ECL assay to measure vaccine response and the Ga and Gb assays to detect RSV infection in pediatric populations [33]. The RSV F, N, Ga and Gb IgG ECL assays described in this study were used to diagnose recent RSV infection in elderly patients using acute and convalescent serum samples collected during a period of respiratory illness. This serological approach may provide a complimentary method to diagnosing RSV infection in elderly patients by RT-PCR or viral culture [7]. Adults are known to shed lower amounts of virus and for shorter windows of time than younger patients, rendering diagnosis of RSV infection using RT-PCR or viral culture difficult if the sample is not collected during the window of viral shedding [47]. Although serologic testing does not offer real-time diagnosis, it is well-suited to the defined sample collection schedules of epidemiology studies or clinical trials. The performance and demonstrated clinical utility of the RSV F, N, Ga and Gb ECL assays suggest that a multiplex serology assay containing these antigens would improve efficiency during clinical sample testing by decreasing the required sample volumes and reducing time and labor costs. Results from development and qualification experiments of a multiplex serology assay using MSD’s ECL technology will be reported shortly.

## Supporting Information

S1 Fig **Titration of motavizumab using an (A) RSV F IgG ECL assay or an (B) RSV F/G IgG diagnostic ELISA.** Four concentrations of motavizumab were serially diluted and added to MSD or ELISA plates. MSD RLU or OD (optical density) values were log_10_-transformed and plotted versus the log_10_-transformed concentration of motavizumab (ng/ml). The upper and lower limits of the linear range were calculated using a 4-parameter logistic fit and marked by dashed red lines.
(TIF)

S2 Fig Performance trending of the assay control. RSV F IgG (n = 323, 14.4% CV); RSV N IgG (n = 66, 12.8% CV); RSV Ga IgG (n = 209, 19.3% CV); RSV Gb IgG (n = 391, 18.5% CV).
(TIF)
